# AI in spotting high-risk characteristics of medical imaging and molecular pathology

**DOI:** 10.1093/pcmedi/pbab026

**Published:** 2021-12-04

**Authors:** Chong Zhang, Jionghui Gu, Yangyang Zhu, Zheling Meng, Tong Tong, Dongyang Li, Zhenyu Liu, Yang Du, Kun Wang, Jie Tian

**Affiliations:** Department of Big Data Management and Application, School of International Economics and Management, Beijing Technology and Business University, Beijing 100048, China; CAS Key Laboratory of Molecular Imaging, Institute of Automation, Chinese Academy of Sciences, Beijing 100190, China; CAS Key Laboratory of Molecular Imaging, Institute of Automation, Chinese Academy of Sciences, Beijing 100190, China; School of Artificial Intelligence, University of Chinese Academy of Sciences, Beijing 100049, China; CAS Key Laboratory of Molecular Imaging, Institute of Automation, Chinese Academy of Sciences, Beijing 100190, China; School of Artificial Intelligence, University of Chinese Academy of Sciences, Beijing 100049, China; CAS Key Laboratory of Molecular Imaging, Institute of Automation, Chinese Academy of Sciences, Beijing 100190, China; School of Artificial Intelligence, University of Chinese Academy of Sciences, Beijing 100049, China; CAS Key Laboratory of Molecular Imaging, Institute of Automation, Chinese Academy of Sciences, Beijing 100190, China; School of Artificial Intelligence, University of Chinese Academy of Sciences, Beijing 100049, China; CAS Key Laboratory of Molecular Imaging, Institute of Automation, Chinese Academy of Sciences, Beijing 100190, China; School of Artificial Intelligence, University of Chinese Academy of Sciences, Beijing 100049, China; CAS Key Laboratory of Molecular Imaging, Institute of Automation, Chinese Academy of Sciences, Beijing 100190, China; School of Artificial Intelligence, University of Chinese Academy of Sciences, Beijing 100049, China; CAS Key Laboratory of Molecular Imaging, Institute of Automation, Chinese Academy of Sciences, Beijing 100190, China; School of Artificial Intelligence, University of Chinese Academy of Sciences, Beijing 100049, China; CAS Key Laboratory of Molecular Imaging, Institute of Automation, Chinese Academy of Sciences, Beijing 100190, China; School of Artificial Intelligence, University of Chinese Academy of Sciences, Beijing 100049, China; CAS Key Laboratory of Molecular Imaging, Institute of Automation, Chinese Academy of Sciences, Beijing 100190, China; School of Artificial Intelligence, University of Chinese Academy of Sciences, Beijing 100049, China; Beijing Advanced Innovation Center for Big Data-Based Precision Medicine, School of Medicine and Engineering, Beihang University, Beijing 100191, China

**Keywords:** machine learning, radiomics, medical imaging, pathology, artificial intelligence

## Abstract

Medical imaging provides a comprehensive perspective and rich information for disease diagnosis. Combined with artificial intelligence technology, medical imaging can be further mined for detailed pathological information. Many studies have shown that the macroscopic imaging characteristics of tumors are closely related to microscopic gene, protein and molecular changes. In order to explore the function of artificial intelligence algorithms in in-depth analysis of medical imaging information, this paper reviews the articles published in recent years from three perspectives: medical imaging analysis method, clinical applications and the development of medical imaging in the direction of pathological molecular prediction. We believe that AI-aided medical imaging analysis will be extensively contributing to precise and efficient clinical decision.

## Introduction

Medical imaging provides clinicians with comprehensive perspectives and rich information. It plays a vital role in disease screening, diagnosis, treatment selection and prognostic evaluation. The changes in the morphology or function of the lesion are determined by many factors such as the patient's individual genes, cells, physiological microenvironment, living habits and living environment. Through data mining technology in conventional imaging diagnosis, the deep characteristics of diseases can be found, reflecting the changes of human tissues, cells and genes, which will have a significant promotion for clinical precision medicine. Since medical imaging can comprehensively, non-invasively and quantitatively observe the overall tumor morphology, and monitor the development process and treatment response of the tumor at any time, it provides a reliable solution to the problem of tumor heterogeneity. Compared with traditional clinical medicine, which only interprets medical images from a visual level, radiomics can dig deeper into the biological features of images and provide clinical decision support.

Radiomics believes that the macroscopic imaging characteristics of tumors are closely related to microscopic gene, protein and molecular changes. Common medical imaging methods currently in clinical practice include computed tomography (CT), magnetic resonance imaging (MRI), ultrasound and positron emission computed tomography (PET). The above-mentioned imaging methods provide a wealth of disease information for clinical diagnosis. Among them, CT scans a thick layer with an X-ray beam and the detector can take a cross-sectional or three-dimensional image of the inspected part. MRI generates magnetic resonance phenomena by applying radio frequency pulses to the human body in a static magnetic field and realizes imaging through processes such as MR signal reception, spatial encoding, and image reconstruction. US scans the human body with ultrasonic sound beams and obtains images of internal organs by receiving and processing reflected signals. PET labels materials with short-lived radionuclides (18F, 11C, etc.), releasing positrons during the decay process and generating opposite photons. Captured by a highly sensitive photon camera and processed by a computer, a three-dimensional image of the aggregation in the organism can be obtained. Besides these non-invasive medical imaging methods, pathological examination is another important technique for clinical analysis and is the gold standard for judging the state of sampled tissues. Advances in equipment have made it possible to preserve and transmit the whole-slide pathological images, therefore promoting the application of artificial intelligent (AI) in pathological image analysis.

The large-scale application of AI technology provides an opportunity for in-depth analysis of medical images. The advantage of AI is that it can use more accurate and generalized models to capture subtle features, powerful classification and prediction capabilities, and provide the possibility to predict microscopic pathology for medical image analysis. AI combined with medical imaging further expands the analysis function of radiomics. Gradually, the macroscopic judgment of the lesion as simply benign or malignant is developing towards the microscopic prediction of molecular typing; the disease state analysis is developing in the direction of gene mutation prediction; the non-invasive imaging prediction is approaching the results of pathological analysis. AI and medical imaging together provide an effective way for precision medicine and is expected to provide a reliable auxiliary analysis method for clinical decision-making.

In the following, this paper will review the articles published in recent years from three perspectives: medical imaging analysis method, clinical applications and the development of medical imaging in the direction of pathological molecular prediction. The second part focuses on the main methods of medical imaging analysis. In the third part, it introduces the application of CT, MRI, PET, Ultrasound and pathological examination, and analyzes the application scope and effect. The fourth part is a summary and prospect of the existing technology and application. It is hoped that this article can provide field overview and research ideas for researchers in related fields.

## Methodology of medical imaging analysis

### Methodological framework

The development of medical imaging analysis is based on the assumption that detailed information can be extracted, thereby providing useful diagnosis information for disease prediction.^1–3^

**Figure 1.**
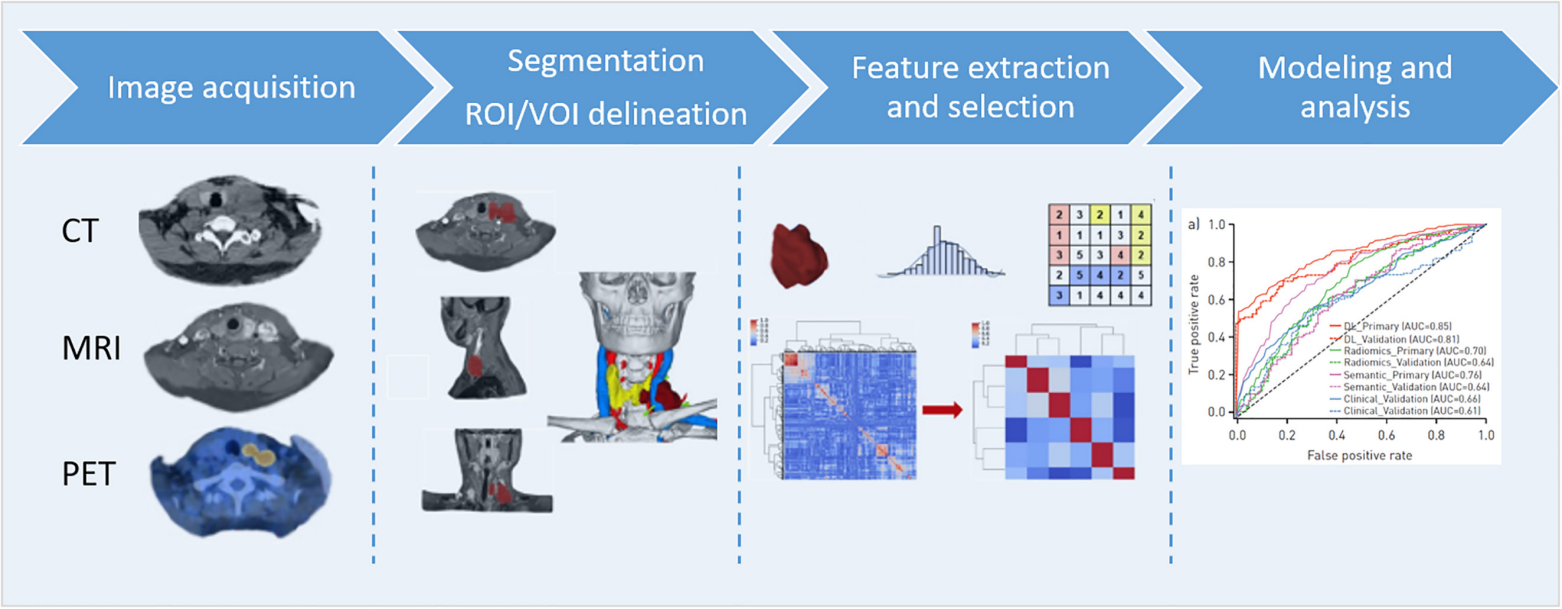
Typical radiomics workflow.

Typical radiomics workflow includes four steps: 1) medical image acquisition; 2) segmentation, areas of interest (ROI) delineation; 3) data cleaning and data enhancement, feature extraction and selection; and 4) modeling and analysis (Fig. 1).^4^ The first important step is to obtain high-quality medical images with uniform standards. Ideally, image resolution, uniform collection parameters, imaging parameters, and others all need to be standardized.^5–7^ In the second step of feature division, target areas (ROI) need to be delineated. The method of segmentation can be manual, semi-automatic, or automated execution. The manual method needs to rely on experienced doctors to divide one by one, and the workload is large. Semi-automatic is a combination of manpower and machine. It requires experienced doctors to identify and modify the boundaries of automatic separation, saving a part of manpower. The automated method does not require human involvement and is more suitable for processing large data sets. The third step is feature extraction. Feature extraction can include shape and geometry features of the ROI area; texture features; intensity features, in which the density distribution reflects tumor heterogeneity. Medical imaging features can also be combined with clinical characteristics, such as clinical and pathological staging, etc. The fourth step of model construction and analysis is to establish a corresponding feasible model.

### Traditional method

Traditional algorithms are often in three steps: medical image segmentation, feature extraction and model construction. Segmentation algorithm is mainly applied for automatic ROI delineation.

Feature extraction and selection directly affect the analysis results of the model. For clinical application, features are usually composed of two parts, including the clinical characteristics and the radiomics features (Table 1). The effect and repeatability of feature extraction from medical images affects the construction of radiomics model. The extracted features are mainly morphological characteristics, including volume, diameter, texture and several transformation functions. The feature evaluation method can be statistics, which evaluates the distribution of gray values or the irregularity of the area; also based on transformation, and the spatial information is converted into frequency.^8^

**Table 1.** Classification of clinical characteristics and radiomics features.

Classification of basic feature	Common features	Statistic feature
Clinical characteristics	Age, BMI, Sex, clinical dementia rating, histological type, clinical staging	Statistics and partition representation
Radiomics features^4^	Volume, diameter, size, shape, location	Histogram statistics
	Texture	Gray-level co-occurrence matrix (GLCM)Gray-level neighborhood difference matrix (GLNDM)Gray-level run length matrix (GLRLM)Gray-level size zone (GLSZM)
	Image feature	Fourier, Gabor, Wavelet and Laplacian transforms

The construction of medical imaging analysis model depends on the clinical problem to be solved and the function to be realized. For classification and prediction, the commonly used methods are statistical and machine learning methods. The commonly used machine learning methods include principal component analysis (PCA), decision tree, random forests (RF), Logistic regression, support vector machine (SVM), least absolute shrinkage and selection operator (LASSO) method, etc.^9–13^

### Deep learning method

Convolutional neural network (CNN) has now been used extensively in medical image classification.^14^ The mostly commonly used models include LeNet,^15^ Alexnet,^16^ VGG19,^17^ GoogLeNet^18^ and ResNet.^19^

Nibali *et al*.^20^ fine-tuned a pre-trained ResNet model to evaluate the classification performance of malignant tumors in lung nodules by using deep learning model. The results show that the combination of deep residual learning and transfer learning can achieve higher accuracy of nodule classification. Nishio *et al*.^21^ used VGG16 to extract features from CT images of lung nodules, demonstrating that transfer learning methods outperformed hand-crafted features and traditional machine learning methods in lung cancer. Marentakis *et al*.^22^ found that in the classification of non-small cell lung cancer(NSCLC) into adenocarcinoma(AC) and squamous cell carcinoma(SCC), the use of CNN combined with long short-term memory (LSTM) network (CNN + LSTM accuracy = 0.74, AUC = 0.78) was more effective and would yield better results than the use of CNN only (the best CNN accuracy = 0.67, AUC = 0.74). An *et al*.^23^ used CNN to extract high-risk features of images. Dual-energy computed tomography (DECT) is a new imaging technique that enables X-ray attenuation data to be obtained at two different energy levels, 100 and 150 keV. Their method uses CNN to extract image features at 100 keV, 150 keV and virtual monoenergetic images (VMI) at 40 keV, respectively; the extracted features were concatenated, and six models were built, including VMI 40 keV model, 100 keV model, 150 keV model, 100 + 150 keV model, 100 + 150 keV and clinical + 100 + 150 keV model. The dataset consisted of 148 patients and was divided into two groups. The first 113 patients were used to train the network and the remaining 35 patients were used to test the performance of the model. The results showed that the model combining 100 + 150 keV and clinical data predicted the most accurate results.

Deep learning has been applied in the clinical diagnosis of many tumors and has shown beneficial effect. The AI method benefits from the technological advancement of computer hardware (graphics processing unit, GPU), and its computational timeliness is also sharply improved.^24,25^ At the same time, more and more open-source and effective calculation framework codes and pre-training models also provide more evolutionary steps for the algorithm iteration to the AI of medical images. In the work of Zheng *et al*., 584 breast cancer patients were enrolled, and ultrasound images were collected. The prediction of axillary lymph node status was realized by radiomics model based on deep learning, with prediction AUC of 0.902.^26^ Marentakis *et al*. Enrolled 102 lung cancer patients using LSTM and Inception model for histological classification analysis, with an AUC of 0.78.^22^ To predict EGFR mutation status in pulmonary adenocarcinoma, Zhao *et al*. used deep learning analysis method based on 3D DenseNets and the AUC reached 0.75.^27^ Similarly, for the prediction of EGFR, Wang *et al*. enrolled 844 patients with lung adenocarcinoma and improved AUC to 0.81 by using the self-built deep learning model.^28^ This noninvasive prediction of EGFR is beneficial for targeted therapy. Yan *et al*. applied bayesian regularization nueral networks to the prediction of IDH and TERT status with 357 glioma patients.^29^ Burlingame *et al*. proposed a deep learning system named ‘speedy histopathological-to-immunofluorescent translation (SHIFT)^30^ which used cycle GAN algorithm^31^ to learn the spatial pattern of paired hematoxylin and eosin (H&E) and immunohistochemistry IHC images. The model is capable of making predictions of DAPI, α-SMA and PanCK distribution using H&E stained images as inputs. This work further demonstrated the capacity of artificial intelligence in histological analysis tasks.

## Applications of medical imaging analysis

**Figure 2.**
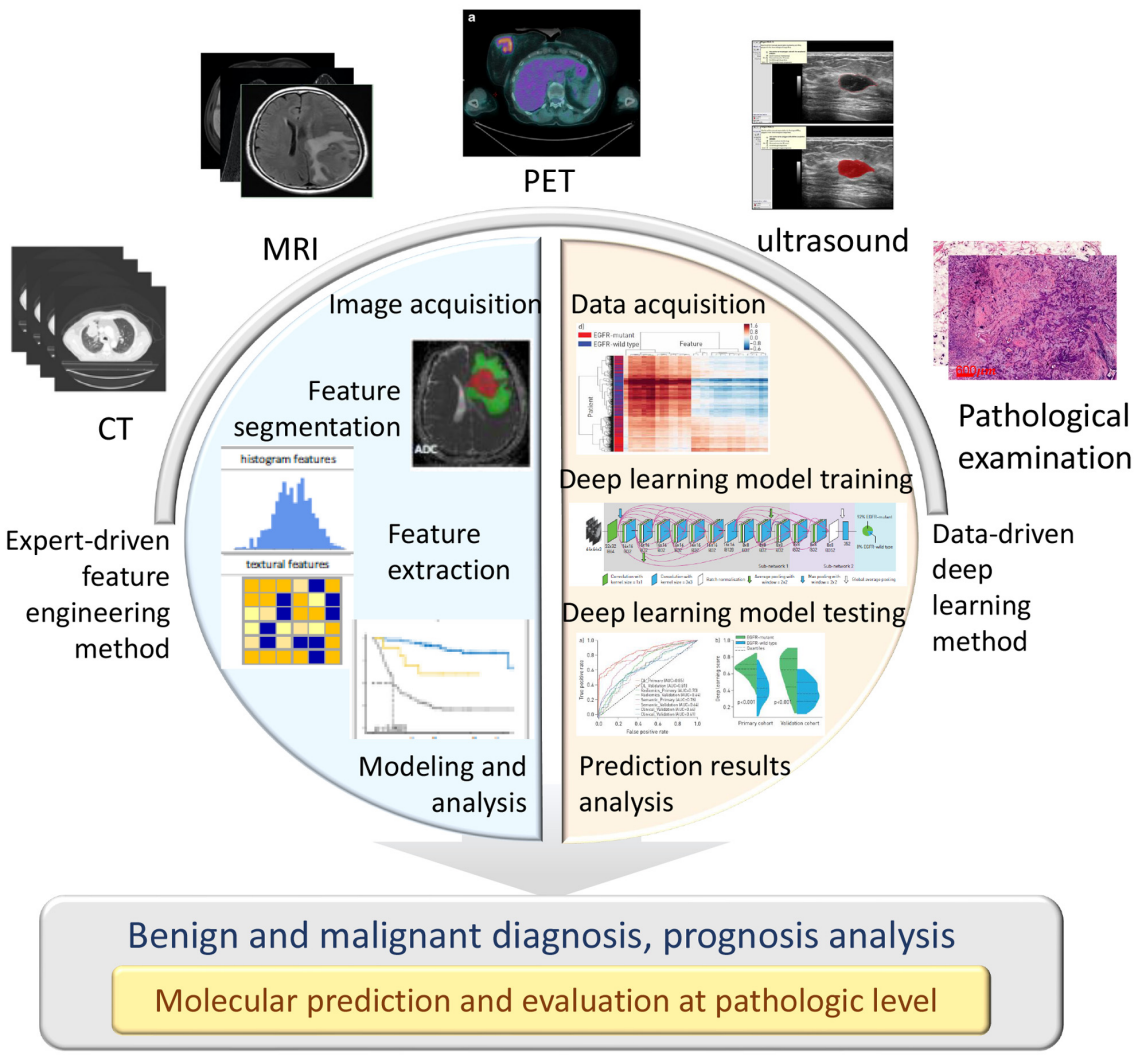
Medical imaging analysis model for pathological and molecular prediction.

We here summarize the use of CT, MRI, PET, ultrasound and pathological examinations, as well as their realization of clinical application and molecular pathological analysis, in diagnosing tumors including breast cancer, hepatocellular tumor, lung cancer, glioma, and others (Fig. 2).

### CT clinical application

Medical images are now used for a wide range of medical applications such as early diagnosis, detection and evaluate patients for treatment in a non-invasive manner.^32^ Among the many imaging modalities, the wide range and high speed of imaging are the characteristics of computed tomography (CT).^33^ CT produces a cross-sectional image of the measurement subject by rotating the X-ray tube with a detector at its relative position to collect the X-ray, using the difference in the attenuation coefficient of the X-ray as they pass through different tissues.^34^

In CT images, the grey level corresponds to the attenuation of X-rays and reflects the proportion of X-rays that are scattered or absorbed.^35^ Given the wealth of detail contained in CT images, their interpretation requires an experienced clinician. Medical staffs can benefit from computer-aided decision-making.^36^ As a result, machine learning and deep learning related to image processing have been widely used in the field of medical imaging analysis (Table 2).^37,38^

**Table 2.** Clinical application of radiomics in molecular and pathological analysis.

Study	Number of patients	Tumor characteristic	Imaging modality	Function and Prediction results	Segmentation and feature selection method/model	Machine learning algorithm
Yang *et al*.^39^	467	lung adenocarcinoma (LADC)	CT	Predicting EGFR mutationAUC 0.789	Nodule segmentation 3D U-net model and pyradiomics	RF
Feng *et al*.^40^	300	Breast cancer	CT	Predicting triple negative breast cancerAUC 0.851	Manual segmentation and LASSO logistic method	Statistics
Ma *et al*.^41^	140	Solid Lung Adenocarcinoma	CT	Predicting AnaplasticLymphoma Kinase Gene RearrangementAUC 0.801	Pearson correlation coefficient and ANOVA or RFE	SVM
Marentakis *et al*.^22^	102	Lung cancer	CT	Histological classificationAUC 0.78	Joint FDG-PET and MRI prediction of lung metastases	LSTM + Inception
Zhang *et al*.^42^	420	lung adenocarcinoma	CT	Predicting EGFR mutation statusAUC 0.835	LASSO and Wilcoxon test, DT, logistic regression	SVM
Wu *et al*.^43^	74	hepatocellular carcinoma	CT	Predicting the Ki-67 marker index	Statistics	Logistic regression
Zhao *et al*.^27^	579	pulmonary adenocarcinoma	CT	Predicting EGFR mutation statusAUC 0.75	Manual delineate	3D DenseNets
Li *et al*.^44^	207	colon cancer	CT	Predicting perineural invasion and KRAS mutationAUC 0.793 and 0.862	Manual delineate	SVM
Wang *et al*.^28^	844	lung adenocarcinoma	CT	Predicting EGFR mutation statusAUC 0.81	A cubic ROI containing the entire tumour manual select	Deep learning model
Sutton *et al*.^45^	273	breast cancer	MRI	Classifying pathologic response post-neoadjuvant chemotherapyAUC 0.83	GMMGLMNet-RF-RFE	Statistics
Fan *et al*.^46^	144	Breast Cancer	MRI	Predicting histological grade and Ki-67 expression levelAUC 0.814 and 0.810	Spatial fuzzy C-means algorithm refined by a Markov random field	Multitask learning method
Shofty *et al*.^47^	47	low-grade gliomas	MRI	1p/19q codeletion status predictionAUC 0.87	AnalyzeDirect software segmentation	Ensemble Bagged Trees
Park *et al*.^48^	121	low-grade gliomas	MRI	Predicting molecular features of glioblastoma in Isocitrate Dehydrogenase Wild-TypeAUC 0.863	Clinical feature + VASARI + radiomics feature	RFESVM
Yan *et al*.^29^	357	glioma	MRI	Predicting IDH and TERT statusAUC 0.884 and 0.669	WaveletLASSO	Bayesian-regularization neural networks
Wu *et al*.^49^	126	diffuse gliomas	MRI	Predicting isocitrate dehydrogenase genotypeAUC 0.931	Automated segmentation	RF
Braman *et al*.^50^	117	Breast cancer	MRI	Predicting pathological complete response to neoadjuvant chemotherapyAUC 0.74	A combined intratumoral and peritumoral radiomics approach	Cluster
Niu *et al*.^51^	182	High-Grade Gliomas	MRI	Estimating the IDH1 GenotypeAUC 0.86	Statistics	LASSO
Umutlu *et al*.^52^	124	Breast cancer	PET/MRI	Breast Cancer Phenotyping and Tumor Decoding	Statistics	LASSO
Zheng *et al*.^26^	584	Breast cancer	US	Predicting axillary lymphnode statusAUC 0.902	Deep learning radiomics model	Deep learning radiomics model

ANOVA, analysis of variance; DT, decision tree; RF, random forest; LASSO, least absolute shrinkage and selection operator; RFE, recursive feature elimination; SVM, support vector machine.

### MRI clinical application

**Figure 3.**
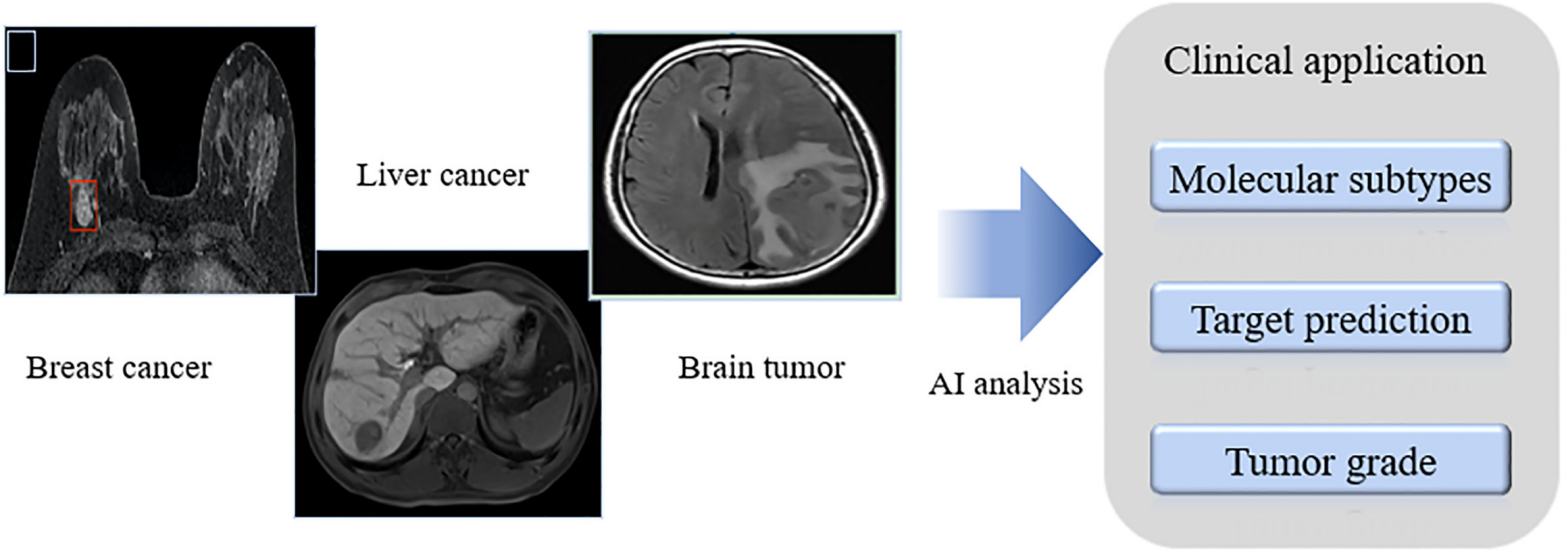
MRI radiomics applied at the molecular level of various tumors.

Recent studies have shown significant heterogeneity in tumor lesions, including variability between tumors and within the same tumor. MRI is a more specific and sensitive method for lesion identification and diagnosis among all available tools because of its higher image resolution. However, for the diagnosis and prediction of tumor at the molecular level, there are limitations in MRI images observed by the naked eye alone. Fortunately, radiomics is increasingly used in tumor diagnosis, prognosis, and treatment selection. The potential of radiomics in precision medicine practice is further enhanced by the ability to consistently acquire microscopic features of medical images that are not visible to the naked eye of human experts (Fig. 3).

The application of MRI radiomics in molecular level of breast cancer (BC) mainly refers to the prediction of molecular subtype. Hormonal status of BC was analyzed by immunohistochemistry (IHC) and divided into three molecular subgroups: HR+ and HER2-, HER2+, and triple negative.^53–55^ Various molecular subtypes have been shown to correlate with treatment planning and prognosis. Among them, IHC only analyzes localized tissue samples of breast cancer, which may not accurately represent the microscopic state of the entire tumor due to the complexity and heterogeneity of the tumor. However, molecular subtypes are confirmed by IHC analysis of sample tissues, which may not reflect the complexity and heterogeneity of the whole tumor. Recent studies have shown that radiomics is expected to be a new imaging label for identifying molecular subtypes of BC patients because of its good performance.^56–62^ Lee *et al*.^57^ found that textural parameters correlated with hormone receptor (HR), HER2 and Ki67 status, and molecular subtypes (p < 0.002). The status of HR and Ki67, grading and molecular subtypes were also correlated with perfusion parameters (*P* < 0.003). Bitencourt *et al*.^58^ used three MRI parameters (two clinical, one radiomic) to achieve the prediction of HER2 burden, then they predicted whether BC patients with HER2+ who received neoadjuvant chemotherapy (NAC) would achieve a pathologically complete response (PCR). One study confirmed that texture features extracted from quantitative ADC map and DCE Map (Flush and Rinse) were able to identify triple negative BC (TNBC) by histogram analysis. The AUC of these models were 0.710 (Luminal A vs. TNBC), 0.763 (HER2+ vs. TNBC) and 0.683 (non-TNBC vs. TNBC), respectively.^63^

The molecular-level prediction tasks of MRI radiomics on hepatocellular carcinoma (HCC) include predicting molecular states related to tumor and immunotherapy. Chen *et al*. proposed a machine learning model based on gadolinium-ethoxybenzyl-diethylenetriamine (Gd-EOB-DTPA)-enhanced MRI to predict the immunoscore related to the density of CD3+ and CD8+.^64^ The AUC of this model based on the selected intratumoral and peritumoral radiomics features and clinical data is up to 0.926. They also illustrated that using combined radiomics features can obtain better predicting performance than only using intratumoral radiomics features. Gu *et al*. established a nomogram model based on ten radiomics features and a clinical characteristics (α-fetoprotein (AFP)) by multivariable logistic regression to predict the Glypican 3 (GPC3) expression which is a biomarker associated with the prognosis of HCC patients.^65^ The imaging sequence used in their research is contrast-enhanced T_1_-weight MRI. This model achieved higher AUCs of 0.926 in training and 0.914 in validation cohorts compared with only using radiomics features. Wang *et al*. incorporated the level of AFP, enhancement patterns of tumor in the arterial phase, irregular margin of tumor, and 11 radiographic features extracted from gadoxetic acid-enhanced MRI images into the final nomogram model to predict the status of cytokeratin (CK) 19 in HCC patients.^66^ The AUCs of this model for predicting CK 19 status can achieve 0.959 and 0.846 in training and validation cohorts. Hectors *et al*. explored the correlation between qualitative or quantitative radiomics features based on contrast-enhanced T1-weighted and diffusion-weighted images.^67^ This study proved that radiomics features are correlated with immunohistochemical cells such as CD3, CD68 and CD31. At protein and mRNA expression level, the radiomics features also have correlation with PD-L1, PD1 and CTLA4.

Noninvasive preoperative grading and detection of key tumor markers in glioma are useful for surgeons. Recent studies have shown that MRI radiomics can extract micropathological features of gliomas from medical images, which may further understand some physiological behavior of gliomas. Studies have found that image fusion models combining radiomic features based on contrast-enhanced T1-weighted imaging (cT1WI) and apparent diffusion coefficient (ADC) achieved AUCs of 0.884 for status of IDH prediction and 0.669 for predicting TERT status. The cT1WI radiomic features performed well with AUC of 0.815 for 1p/19q status prediction.^68^ In addition, this study confirmed that MRI-based radiomics can be used to noninvasively detect molecular populations and predict glioma survival regardless of glioma grade. These findings have been confirmed by other investigators.^69–74^ Yogananda *et al*.^75^ proposed a new deep learning network named MGMT-net that can efficiently identify the status of MGMT promoter methylation based on T2-weighted images (T2WI), with good accuracy of 0.947 in the validation cohort confirmed by tri-fold cross-validation. Akbari *et al*.^76^ constructed a study cohort of 129 patients with new glioblastoma. Then they obtained imaging labels of EGFRvIII from MRI images using radiomics techniques and achieved accuracy of 0.853 and 0.87 for identifying EGFRvIII mutated status in validation and test cohort, confirming that imaging signature of EGFRvIII can reveal a complex and unique macro glioblastoma phenotype.

One common childhood brain tumor is medulloblastoma (MB), which has a very high degree of malignancy. A study by Yan *et al*.^77^ found that clinical and MRI imaging information from routine preoperative examinations could predict molecular subgroups of MB with high accuracy by using a machine learning algorithm. The model showed excellent predictive performance for wingless with an AUC of 0.9097 and accuracy of 0.8, and for sonic hedgehog with an AUC of 0.8654 and accuracy of 0.867. Recent studies have shown that some patients with astrocytoma, the most common type of glioma with a poor prognosis, have improved survival by responding well to temozolomide (TMZ) chemotherapy.^78,79^ The reason is that these patients were grade II-IV astrocytomas with methylation of the oxy-6-methylguanine-DNA methyltransferase.^80,81^ Wei *et al*.^82^ found that in the training and validation cohort, fusion radiomic features exhibited the highest ability in predicting methylation of MGMT promoter, with AUCs of 0.925 and 0.902 respectively. Additionally, MRI radiomics performed well in predicting overall survival for patients who completed TMZ chemotherapy (P = 0.003 for high risk *vs*. low risk). Zormpas-Petridis *et al*.^83^ revealed that MRI-based functional imaging can detect apoptotic responses to MYCN-targeted small-molecule inhibitors in a genetically engineered murine model of MYCN-driven neuroblastoma.

In summary, fusion radiomic features based on MRI images are important for predicting molecular subtype and prognostic analysis of lesions in various malignancies. Current studies have aimed to assess potential associations between tumor microscopic features and MRI radiomic features, but results have not yet been uniform. From the development of various radiomic models to their actual application in clinical practice (such as prediction of histopathological features), more and more extensive studies are necessary.

### PET clinical application

PET is a functional imaging modality that noninvasively shows the metabolic processes of disease *in vivo*. It is widely applied in clinical practice for diagnosis, staging, assessment of therapeutic response, and prediction of gene mutations and prognosis. The introduction of radiomics has stimulated a new platform for non-invasive tissue characterization based on functional imaging. Some studies have predicted clinical outcomes based only on the radiomics of PET images. In the study of Li *et al*.,^84^ radiological characteristics were extracted and evaluated from static images, early summary images, and dynamic ^18^F-FET-PET images to predict TERTp-mutation status in isocitrate dehydrogenase (IDH) gene-wild-type high-grade glioma patients, where recursive feature elimination and logistic regression are used; the study showed that the model based on dynamic ^18^F-FET-PET features could anticipate TERTp-mutation status (AUC 0.82, sensitivity 92.1%). In two other studies also targeting gliomas, they used different radioactive tracers for functional imaging. Qian *et al*.^85^ demonstrated that radiological features of ^18^F-DOPA-PET imaging can be used to predict pathological O^6^-methylguanine methyltransferase status (accuracy 80%±10%). Li *et al*.^86^ used support vector machines to generate ^18^F-FDG-PET imaging features combined with clinical features to predict the potential of IDH genotype status in patients with glioma, and verified the effectiveness of the model.

**Figure 4.**
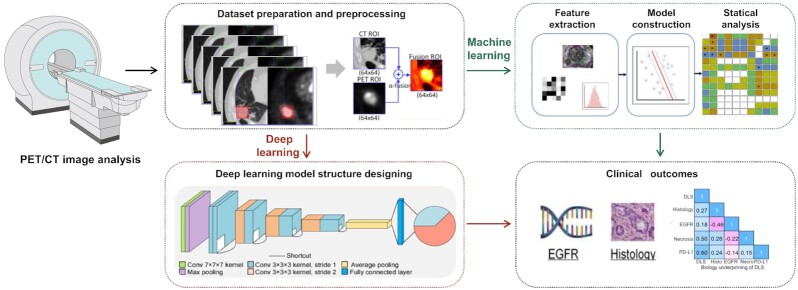
PET combined with CT for medical imaging analysis.

Since PET presents limited details on the morphology of lesions, it is often used in combination with CT (PET/CT, Fig. 4) or MRI (PET/MRI) to obtain both metabolic and structural information. Such multimodal imaging analysis shows good application results.

#### PET/CT-based radiomics applications

The prediction of specific biomarkers in lung cancer is a typical example of PET/CT-based radiomics applications. Mu *et al*. used small residual convolutional networks to analyze ^18^F-FDG-PET/CT images of 697 non-small cell lung cancer (NSCLC) patients from three institutions and to develop deep learning scores to anticipate PD-L1 expression status. Their work was well validated in training, and testing cohorts (AUC ≥ 0.82), which could be used to identify patients with immune checkpoint inhibitor sensitive NSCLC.^87^ Similarly, Zhang *et al*.^88^ used LASSO algorithm to select radiologic features that predict EGFR mutations based on ^18^F-FDG PET/CT images from 248 NSCLC patients. They found that the best prediction for EGFR mutations was with the nomogram developed using a combination of radiological characteristics score and clinical variables (AUC = 0.87). While another study used AI software to extract radiological features from PET/CT images of patients with lung adenocarcinoma, 22 radiological features and 3 clinical features were selected to establish a model which predicted ALK rearrangement status (AUC = 0.88).^89^  ^18^F-PET/CT-based radiomics are also suitable for patients with NSCLC who are preparing for stereotactic body radiation therapy (SBRT), which can predict the potential of circulating tumor cells before and after SBRT to co-guide patients' subsequent treatment.^90^ In addition, artificial intelligence radiomics has made new advances in other malignancies based on PET/CT images. Feature extraction (SUVmax, histogram parameters and texture features) was performed on ^18^F-FDG PET images of 38 non-metastatic luminal breast cancer patients in one study and correlation of these extracted features with progesterone receptor and estrogen receptor expression was observed.^91^ While another study population was early-stage cervical cancer patients, Li *et al*. demonstrated that PET-CT-based radiomics integrating the primary tumor and perineural regions could predict E-cadherin.^92^ Different from the above studies with better predictive value, Saadani *et al*. explored the use of radiomic ^18^F-FDG PET/CT features to predict B-rapidly accelerated fibrosarcoma valine 600 (BRAFV600) mutation status in melanoma patients and explored six different methods of feature selection. The results showed that the AUC for radiomics prediction of BRAFV600 mutation in melanoma patients ranged from 0.54 to 0.62 and are influenced by the feature selection method.^93^

#### PET/MRI-based radiomics applications

A new trend in PET imaging technology introduced in the last few years is the PET-Magnetic Resonance Imaging (PET-MRI) system. This system uses MRI as an alternative to CT to eliminate additional radiation dose and can greatly improve the clarity of soft tissue imaging that CT tissue cannot provide. In Umutlu *et al*.'s study,^52^ the performance of synchronous ^18^F-FDG PET/MRI as a comprehensive radiological platform for breast cancer subtype analysis, hormone receptor status, and proliferation rate was investigated. They used LASSO regression to select the most significant radiological characteristics in ^18^F-FDG PET/MRI images from 124 breast cancer patients. Then, support vector machines were used for five-fold cross-validation to form a prediction model for the combination of various imaging data series. Finally, ^18^F-FDG PET/MRI can be used to obtain morphological, functional and metabolic data simultaneously for comprehensive and high-quality radiomic analysis of breast cancer phenotype and tumor decoding. Zaragori *et al*.^94^ used ^18^F-FET PET-MRI radiomics to non-invasively predict IDH genotype, O^6^-methylguanine methyltransferase promoter methylation status and alpha thalassemia/mental retardation syndrome X-linked (ATRX) genotype with 1p/19q coding deletion, both of which showed good prediction.

#### Four-modalities model

Different from above, Matsui^95^ diagnose the molecular subtype of lower-grade gliomas based deep learning (DL) and multi-modalities analysis. They designed a four-modalities (MRI, PET, CT and clinical information) deep learning model to learn and extract relative features automatically.

### Ultrasound clinical application

**Figure 5.**
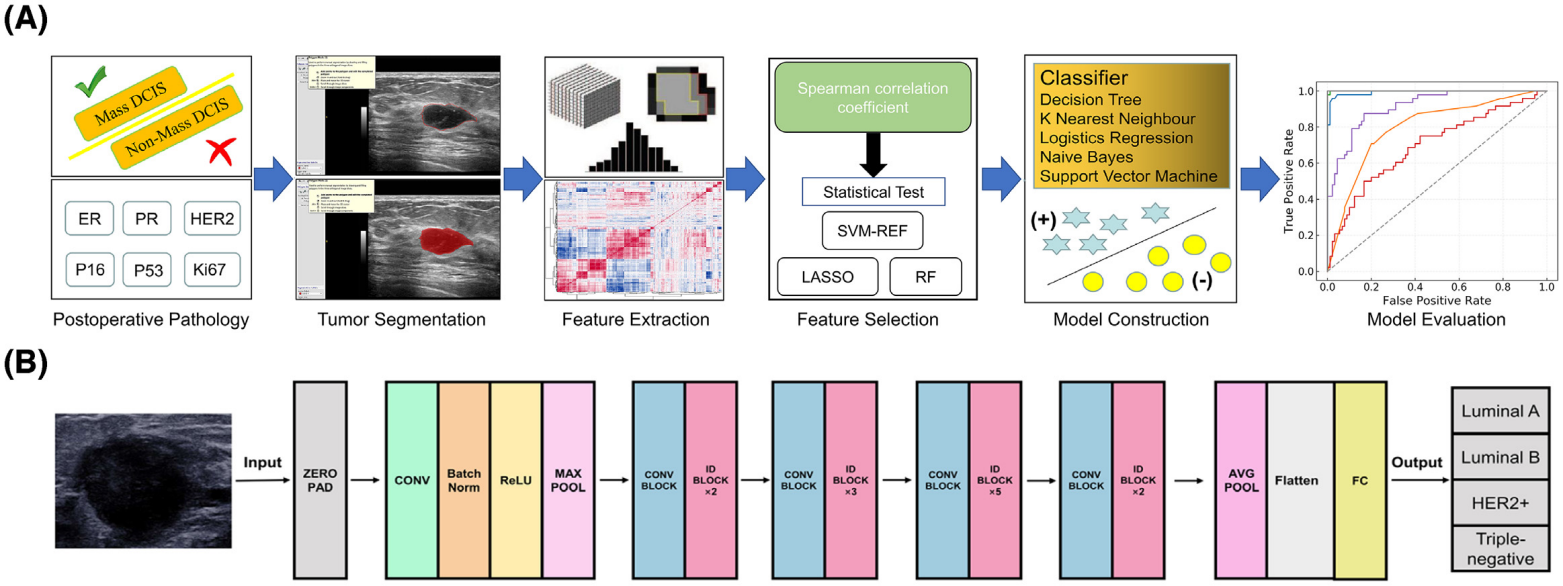
The workflow of (A) machine learning and (B) deep learning ultrasound radiomics for molecular subtype prediction.

Ultrasound imaging is a simple, flexible, low-cost and radiation-free imaging modality, especially suitable for imaging thyroid, breast and liver tissues. However, because ultrasound imaging has a lower resolution than other modalities and is greatly affected by the operator, the image quality is relatively poor and unstable, and the tumor boundary is often not particularly clear. These disadvantages often limit the accuracy of direct analysis of ultrasound images by radiologists. With the development of artificial intelligence, ultrasound radiomics has been widely used in diagnosis and prediction tasks with a good performance. In recent years, ultrasound radiomics has gradually involved cancer-related molecular prediction tasks. The application of ultrasound radiomics at the molecular level is mainly focused on the classification of breast cancer molecular subtypes. Machine learning is also an important technique for feature selection and model development (Fig. 5A). Cui *et al*. used the ultrasound features, according to the fifth edition of Breast Imaging Reporting and Data System (BI-RADS) and the elasticity ultrasound features specified by the WFUMB guidelines to construct Ki67 and P53 expression prediction models.^96^ It was found that the high expression of Ki67 was associated with the loss of echo halo, posterior acoustic enhancement and high BI-RADS category. The high expression of P53 was correlated with the loss of echo halo and high classification of BI-RADS. The AUC of prediction models reached0.78 for Ki67 and 0.71 for P53. The above research only used the clinical appearance features of ultrasound images, ignoring more valuable image features such as grayscale and texture features, thus the prediction accuracy is not high. Wu *et al*. proposed six machine learning models with a series of handcraft image features that are able to predict the expression of multiple breast ductal carcinoma-related molecules including ER, PR, HER2, Ki67, P16 and P53.^97^ The features used in this study contained 5234 image features with mathematical meaning, and different machine learning models for feature selection were used for different molecular prediction tasks. In the end, the AUC of the test cohort was up to 0.84 for ER, 0.78 for PR, 0.74 for HER2, 0.86 for Ki67, 0.78 for P16 and 0.74 for P53 expression prediction. Recently, more and more studies have applied deep learning to the prediction of breast cancer molecular subtypes. Compared with machine learning methods based on manual selected features, deep learning model can adaptively extract high-level advanced features of images that are highly correlated with these molecules through data-driven learning (Fig. 5B). In the case of big data, the prediction performance of molecular expression can be further improved by using deep learning models. Zhang *et al*. proposed one deep learning model for molecular subtype diagnosis.^98^ This study contains a multicenter and big dataset including more than 3 000 ultrasound images and 2 000 patients to train and test the model. The model achieved high performance in identifying different molecular subtypes of breast cancer. The AUCs of the test cohort was 0.864 for identifying triple-negative breast cancer, 0.811 for HER2+ and 0.837 for HR+ breast cancer. Similarly, Jiang *et al*. also developed the deep learning model for breast cancer molecular subtype assessment based on a multicenter dataset with more than 4 828 ultrasound images and 1 275 patients.^99^ This model achieved higher prediction performance, identifying the four breast cancer molecular subtypes including Lumina A, Lumina B, HER2+ and triple-negative, with accuracy ranging from 80.07% to 97.02% and 87.94% to 98.83% in two test cohorts, respectively. Furthermore, this study also proposed an additional deep learning model for distinguishing luminal disease from non-luminal disease. The positive predictive value on the two test cohorts reached 93.29% and 88.21%, respectively. Zheng *et al*. proposed a model by using deep learning method for predicting axillary lymph node status in early-stage breast cancer, with AUC value of 0.902 for lymph node metastasis.^26^

### Pathological examination

Over the past few decades, faster computation capacity and cheaper storage have enabled digital pathology to gain rapid development.^100–105^ Pathologists can study digital pathological images more easily and flexibly than in the past. The past 20 years have seen the beginning and development of digital imaging in pathology, which allows whole slides to be imaged and stored permanently at high resolution. These progresses have facilitated the application of deep learning-based methods in pathological examination analysis. AI is an emerging approach and has been widely applied in medical image analysis, such as nuclei segmentation,^106^ cancer diagnoses^103,107^ and cancer subtype classification.^108^

Christiansen *et al*. proposed a method based on deep learning to predict the distribution of fluorescent markers in unlabeled images by using a deep neural network that trains unlabeled and labeled images in pairs.^109^ The results showed that the fluorescence labeling algorithm based on deep learning can be used to predict the fluorescence labeling of transmitted light images. This demonstrates the potential of the DL-based approach in histological image synthesis tasks. Diao *et al*. proposed a computational pathology process that integrated high-resolution cell-level information from whole-slide images to predict molecular derived phenotypes for five different cancer types.^110^ The proposed approach combines the deep learning method with the interpretability of human interpretable features. It can integrate prior knowledge and achieves the performance of an end-to-end model. Bian *et al*. utilized convolutional neural networks to learn the spatial patterns incorporated within H&E images and make predictions of the distribution of PanCK, DAPI, CD3 and CD20 biomarkers.^111,112^ The proposed works showed that artificial intelligence can be used in biomarker distribution prediction tasks, which can help aid pathologists in image analysis. The applications of AI in pathological examination are shown in Table 3.

**Table 3.** Application of AI in pathological examination.

Application category	Application examples
Diagnosis	Benign and malignant diagnosis^113^Tumor aggressiveness classification^114,115^Tumor differentiation prediction^116^
Efficacy prediction	Tumor regression grade of neoadjuvant therapy prediction^117^
Gene prediction	Classification and mutation prediction from non-small cell lung cancer^118^Microsatellite instability prediction^25^
Biomarker prediction	Prediction of fluorescence label distribution in unlabeled images^109^Predictions of DAPI, α-SMA and PanCK distribution^31^Predictions of the distribution of PanCK, DAPI, CD3 and CD20 biomarkers^111,112^
Prognosis prediction	Survival prediction^119–123^

Artificial intelligence demonstrates exciting potential in the challenge of digital pathological image quantitative analysis and prediction model construction. The method can be summarized into expert-driven feature engineering methods and data-driven deep learning methods.^124,125^ The expert-driven feature engineering method usually includes three steps: artificially defined pathology image feature extraction, feature selection, and modeling.^119,120^ The extracted features are used to quantitatively describe cell-level events such as cell mitosis, multi-nucleation process, and cell subtypes, which was further for the description of spatial architecture and arrangement of cells. After these features are screened, the features are integrated through machine learning, and models are constructed for specific prediction tasks. The data-driven deep learning method employs convolutional neural networks (CNN)^126^ to predict the patch of whole slide image (WSI) end-to-end, and then integrates the prediction results of multiple patches through a voting machine to obtain patient-level prediction results based on several patch-level results.^118,121^ Compared with expert-driven feature engineering method, data-driven deep learning method reduces the dependence on prior pathological knowledge and automatically learns meaningful features from data. In this way, feature design iterations and repeated expert discussions are reduced greatly.^114,124,127^ Currently, AI-based quantitative analysis methods of pathological images have been successfully tried in clinical needs such as diagnosis,^113–116,127^ efficacy prediction,^117^ gene prediction,^25,118^ and prognosis prediction^119,121,122^ of multiple cancers, and have shown good patient benefits. The cancer types involved include breast cancer, prostate cancer, rectal cancer, and many others. The clinical challenges that have been solved involve benign and malignant diagnosis,^113^ tumor aggressiveness classification,^114,115^ tumor differentiation prediction,^116^ microsatellite instability prediction,^25^ tumor regression grade of neoadjuvant therapy prediction^117^ and survival prediction.^119–123^ The AI showed significant improvement in the evaluation of classification accuracy, sensitivity, specificity, and other indicators.^24^ The combination of pathologists' diagnosis results and AI methods can effectively improve the consistency of diagnosis among doctors of different experiences and achieve better performance than doctors in some specific tumor problems.^114,115^ Hopefully, AI is able to provide an effective micro-information reference for precise and personalized treatment, thereby improving patient benefits.

## Discussion and conclusion

Medical imaging provides an important source of clinical information for medical diagnosis, efficacy evaluation, and patient prognosis and survival prediction. With the development of machine learning, artificial intelligent technology can extract key information, which is vulnerable to be missed by human observation, from a large amount of data. The combination of artificial intelligence and medical imaging provides a possible technical means for precision medical analysis. Especially correlation studies have found that pathological and molecular level information is highly correlated with medical imaging features.^128–130^ This allows medical imaging to provide the possibility of molecular-level biological feature diagnosis.

This article mainly analyzes the current research status of medical imaging technology combined with artificial intelligence, especially the systematic research on this method to explore molecular pathology information. The survey results have also proved the feasibility of radiomics to analyze molecular pathology.

However, there are also some limitations and challenges, which require continuous research in the future to further overcome the deficiencies of medical imaging. Specifically, for data acquisition, the protocol and instrument changes between different institutions will reduce the robustness of the radiomics model. Data sharing between institutions involves challenges in patient privacy, which may be a limiting factor in the development of a unified model. This requires standard imaging protocols, repeatable and consistent image processing, and collaboration between agencies to create large amounts of annotated datasets. In addition, it is necessary to design robust imaging features,^131^ and specify unified algorithm design standards and evaluation standards, which will help establish a reliable radiomic model. Furthermore, studies have shown that radiomics, when combined with histopathology, genomics or molecular classification, and immunophenotype, can achieve more precise results in predicting patient prognostic characteristics.^132^

In the future development, medical imaging analysis will focus on data sharing, carefully designed, forward-looking and comprehensive research. It has great potential in helping understanding of the *in vivo* physiology of tumors and provides opportunities for optimizing patient clinical care.^133^ By incorporating more data and making the model more robust, the increasing prediction accuracy of medical imaging analysis will continue to contribute to personalized medicine. Real-time predictive analysis can be obtained from large semi-automatic patient data sets and electronic medical records to provide insights into various disease processes. In conclusion, in the new era of precision medicine, AI-aided medical imaging analysis will be extensively contributing to clinical decision.
